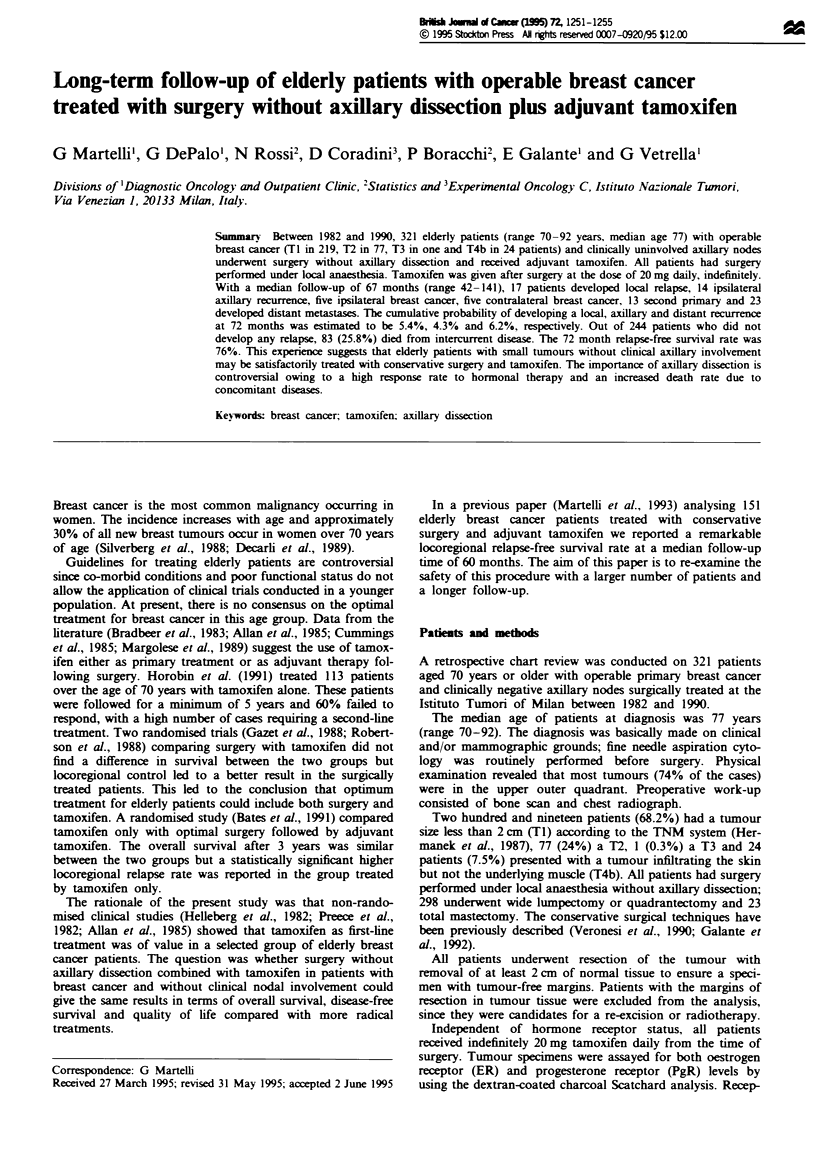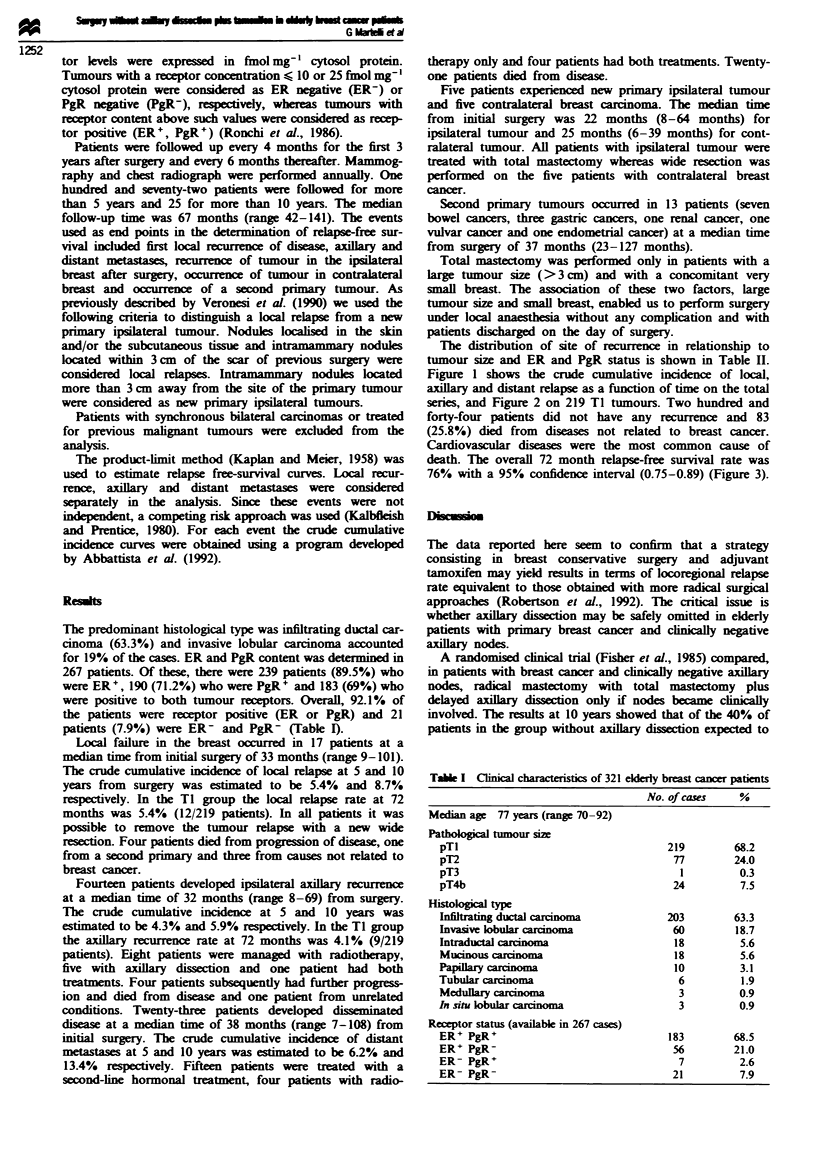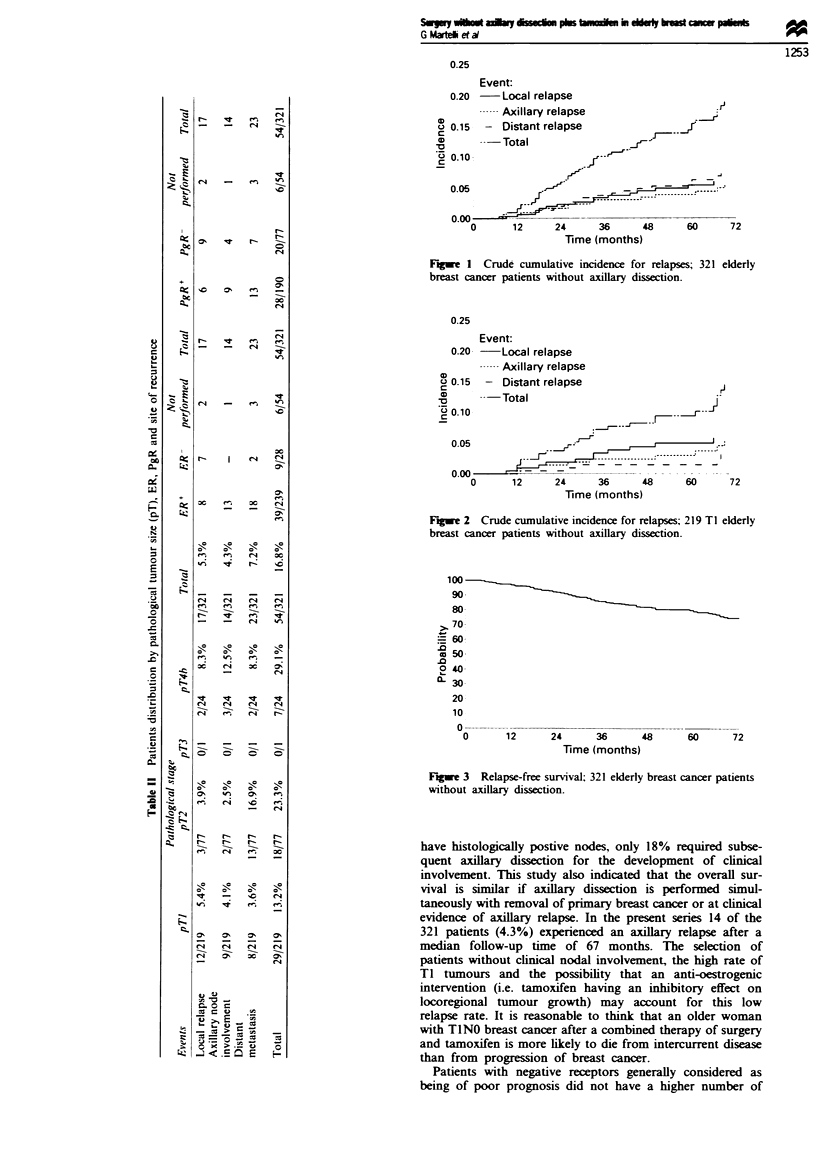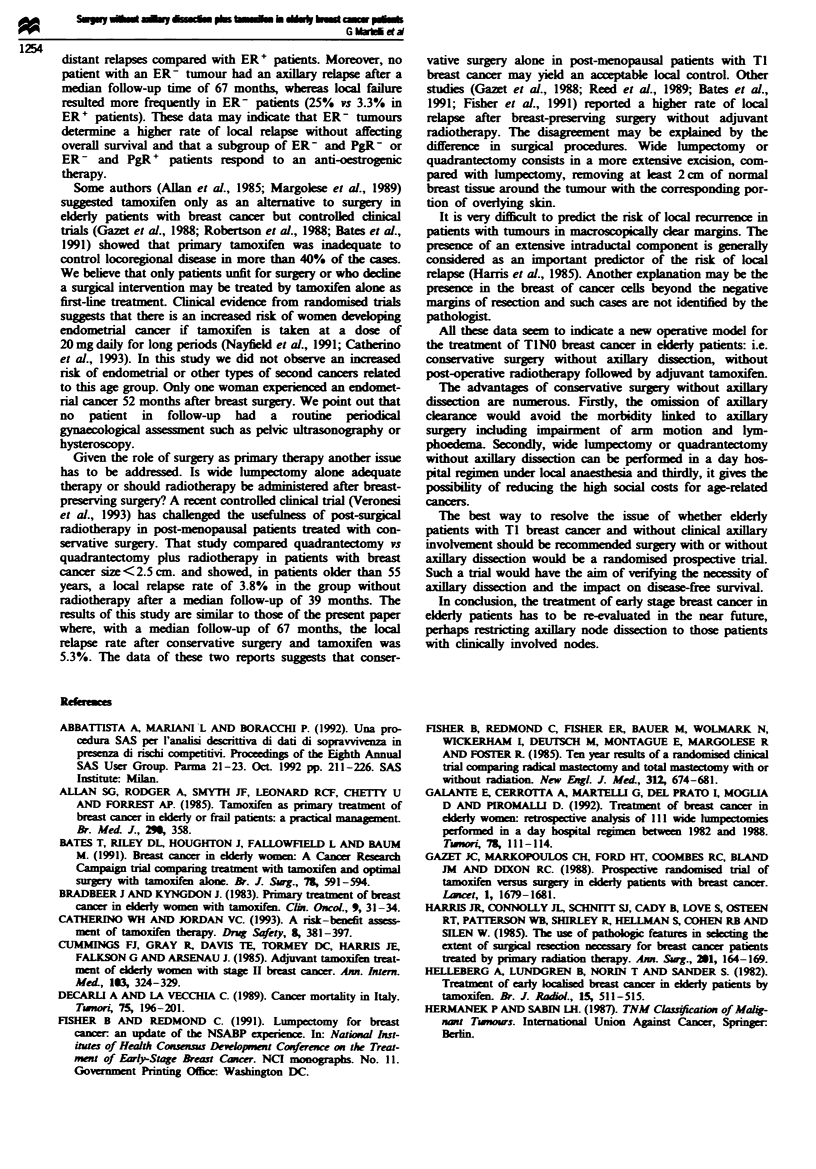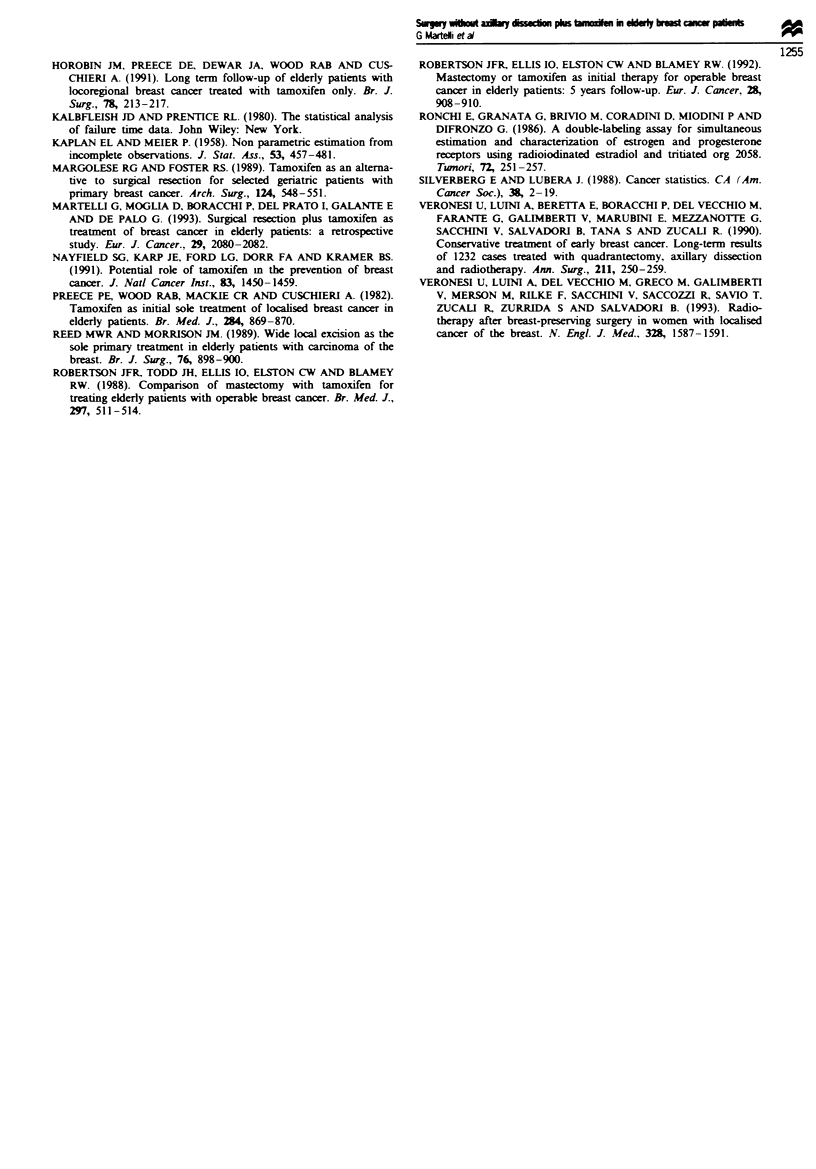# Long-term follow-up of elderly patients with operable breast cancer treated with surgery without axillary dissection plus adjuvant tamoxifen.

**DOI:** 10.1038/bjc.1995.495

**Published:** 1995-11

**Authors:** G. Martelli, G. DePalo, N. Rossi, D. Coradini, P. Boracchi, E. Galante, G. Vetrella

**Affiliations:** Division of Diagnostic Oncology and Outpatient Clinic, Istituto Nazionale Tumori, Milan, Italy.

## Abstract

Between 1982 and 1990, 321 elderly patients (range 70-92 years, median age 77) with operable breast cancer (T1 in 219, T2 in 77, T3 in one and T4b in 24 patients) and clinically uninvolved axillary nodes underwent surgery without axillary dissection and received adjuvant tamoxifen. All patients had surgery performed under local anaesthesia. Tamoxifen was given after surgery at the dose of 20 mg daily, indefinitely. With a median follow-up of 67 months (range 42-141), 17 patients developed local relapse, 14 ipsilateral axillary recurrence, five ipsilateral breast cancer, five contralateral breast cancer, 13 second primary and 23 developed distant metastases. The cumulative probability of developing a local, axillary and distant recurrence at 72 months was estimated to be 5.4%, 4.3% and 6.2%, respectively. Out of 244 patients who did not develop any relapse, 83 (25.8%) died from intercurrent disease. The 72 month relapse-free survival rate was 76%. This experience suggests that elderly patients with small tumours without clinical axillary involvement may be satisfactorily treated with conservative surgery and tamoxifen. The importance of axillary dissection is controversial owing to a high response rate to hormonal therapy and an increased death rate due to concomitant diseases.


British Journal of Cancer (1995) 72, 1251-1255

© 1995 Stockton Press All rights reserved 0007-0920/95 $12.00

### Long-term follow-up of elderly patients with operable breast cancer treated with surgery without axillary dissection plus adjuvant tamoxifen

G Martelli[1], G DePalo[1], N Rossi[2], D Coradini[3], P Boracchi[2], E Galante[1] and G Vetrella[1]

*Divisions of [1]Diagnostic Oncology and Outpatient Clinic, [2]Statistics and [3]Experimental Oncology C, Istituto Nazionale Tumori, Via Venezian 1, 20133 Milan, Italy.*

**Summary** Between 1982 and 1990, 321 elderly patients (range 70–92 years, median age 77) with operable breast cancer (T1 in 219, T2 in 77, T3 in one and T4b in 24 patients) and clinically uninvolved axillary nodes underwent surgery without axillary dissection and received adjuvant tamoxifen. All patients had surgery performed under local anaesthesia. Tamoxifen was given after surgery at the dose of 20 mg daily, indefinitely. With a median follow-up of 67 months (range 42–141), 17 patients developed local relapse, 14 ipsilateral axillary recurrence, five ipsilateral breast cancer, five contralateral breast cancer, 13 second primary and 23 developed distant metastases. The cumulative probability of developing a local, axillary and distant recurrence at 72 months was estimated to be 5.4%, 4.3% and 6.2%, respectively. Out of 244 patients who did not develop any relapse, 83 (25.8%) died from intercurrent disease. The 72 month relapse-free survival rate was 76%. This experience suggests that elderly patients with small tumours without clinical axillary involvement may be satisfactorily treated with conservative surgery and tamoxifen. The importance of axillary dissection is controversial owing to a high response rate to hormonal therapy and an increased death rate due to concomitant diseases.

**Keywords:** breast cancer; tamoxifen; axillary dissection

Breast cancer is the most common malignancy occurring in women. The incidence increases with age and approximately 30% of all new breast tumours occur in women over 70 years of age (Silverberg *et al.*, 1988; Decarli *et al.*, 1989).

Guidelines for treating elderly patients are controversial since co-morbid conditions and poor functional status do not allow the application of clinical trials conducted in a younger population. At present, there is no consensus on the optimal treatment for breast cancer in this age group. Data from the literature (Bradbeer *et al.*, 1983; Allan *et al.*, 1985; Cummings *et al.*, 1985; Margolese *et al.*, 1989) suggest the use of tamoxifen either as primary treatment or as adjuvant therapy following surgery. Horobin *et al.* (1991) treated 113 patients over the age of 70 years with tamoxifen alone. These patients were followed for a minimum of 5 years and 60% failed to respond, with a high number of cases requiring a second-line treatment. Two randomised trials (Gazet *et al.*, 1988; Robertson *et al.*, 1988) comparing surgery with tamoxifen did not find a difference in survival between the two groups but locoregional control led to a better result in the surgically treated patients. This led to the conclusion that optimum treatment for elderly patients could include both surgery and tamoxifen. A randomised study (Bates *et al.*, 1991) compared tamoxifen only with optimal surgery followed by adjuvant tamoxifen. The overall survival after 3 years was similar between the two groups but a statistically significant higher locoregional relapse rate was reported in the group treated by tamoxifen only.

The rationale of the present study was that non-randomised clinical studies (Helleberg *et al.*, 1982; Preece *et al.*, 1982; Allan *et al.*, 1985) showed that tamoxifen as first-line treatment was of value in a selected group of elderly breast cancer patients. The question was whether surgery without axillary dissection combined with tamoxifen in patients with breast cancer and without clinical nodal involvement could give the same results in terms of overall survival, disease-free survival and quality of life compared with more radical treatments.

Correspondence: G Martelli
Received 27 March 1995; revised 31 May 1995; accepted 2 June 1995

In a previous paper (Martelli *et al.*, 1993) analysing 151 elderly breast cancer patients treated with conservative surgery and adjuvant tamoxifen we reported a remarkable locoregional relapse-free survival rate at a median follow-up time of 60 months. The aim of this paper is to re-examine the safety of this procedure with a larger number of patients and a longer follow-up.

### Patients and methods

A retrospective chart review was conducted on 321 patients aged 70 years or older with operable primary breast cancer and clinically negative axillary nodes surgically treated at the Istituto Tumori of Milan between 1982 and 1990.

The median age of patients at diagnosis was 77 years (range 70–92). The diagnosis was basically made on clinical and/or mammographic grounds; fine needle aspiration cytology was routinely performed before surgery. Physical examination revealed that most tumours (74% of the cases) were in the upper outer quadrant. Preoperative work-up consisted of bone scan and chest radiograph.

Two hundred and nineteen patients (68.2%) had a tumour size less than 2 cm (T1) according to the TNM system (Hermanek *et al.*, 1987), 77 (24%) a T2, 1 (0.3%) a T3 and 24 patients (7.5%) presented with a tumour infiltrating the skin but not the underlying muscle (T4b). All patients had surgery performed under local anaesthesia without axillary dissection; 298 underwent wide lumpectomy or quadrantectomy and 23 total mastectomy. The conservative surgical techniques have been previously described (Veronesi *et al.*, 1990; Galante *et al.*, 1992).

All patients underwent resection of the tumour with removal of at least 2 cm of normal tissue to ensure a specimen with tumour-free margins. Patients with the margins of resection in tumour tissue were excluded from the analysis, since they were candidates for a re-excision or radiotherapy.

Independent of hormone receptor status, all patients received indefinitely 20 mg tamoxifen daily from the time of surgery. Tumour specimens were assayed for both oestrogen receptor (ER) and progesterone receptor (PgR) levels by using the dextran-coated charcoal Scatchard analysis. Receptor levels were expressed in fmol $mg^{-1}$ cytosol protein. Tumours with a receptor concentration ≤ 10 or 25 fmol $mg^{-1}$ cytosol protein were considered as ER negative ($ER^-$) or PgR negative ($PgR^-$), respectively, whereas tumours with receptor content above such values were considered as receptor positive ($ER^+$, $PgR^+$) (Ronchi *et al.*, 1986).

Patients were followed up every 4 months for the first 3 years after surgery and every 6 months thereafter. Mammography and chest radiograph were performed annually. One hundred and seventy-two patients were followed for more than 5 years and 25 for more than 10 years. The median follow-up time was 67 months (range 42–141). The events used as end points in the determination of relapse-free survival included first local recurrence of disease, axillary and distant metastases, recurrence of tumour in the ipsilateral breast after surgery, occurrence of tumour in contralateral breast and occurrence of a second primary tumour. As previously described by Veronesi *et al.* (1990) we used the following criteria to distinguish a local relapse from a new primary ipsilateral tumour. Nodules localised in the skin and/or the subcutaneous tissue and intramammary nodules located within 3 cm of the scar of previous surgery were considered local relapses. Intramammary nodules located more than 3 cm away from the site of the primary tumour were considered as new primary ipsilateral tumours.

Patients with synchronous bilateral carcinomas or treated for previous malignant tumours were excluded from the analysis.

The product-limit method (Kaplan and Meier, 1958) was used to estimate relapse free-survival curves. Local recurrence, axillary and distant metastases were considered separately in the analysis. Since these events were not independent, a competing risk approach was used (Kalbfleish and Prentice, 1980). For each event the crude cumulative incidence curves were obtained using a program developed by Abbattista *et al.* (1992).

### Results

The predominant histological type was infiltrating ductal carcinoma (63.3%) and invasive lobular carcinoma accounted for 19% of the cases. ER and PgR content was determined in 267 patients. Of these, there were 239 patients (89.5%) who were $ER^+$, 190 (71.2%) who were $PgR^+$ and 183 (69%) who were positive to both tumour receptors. Overall, 92.1% of the patients were receptor positive (ER or PgR) and 21 patients (7.9%) were $ER^-$ and $PgR^-$ (Table I).

Local failure in the breast occurred in 17 patients at a median time from initial surgery of 33 months (range 9–101). The crude cumulative incidence of local relapse at 5 and 10 years from surgery was estimated to be 5.4% and 8.7% respectively. In the T1 group the local relapse rate at 72 months was 5.4% (12/219 patients). In all patients it was possible to remove the tumour relapse with a new wide resection. Four patients died from progression of disease, one from a second primary and three from causes not related to breast cancer.

Fourteen patients developed ipsilateral axillary recurrence at a median time of 32 months (range 8–69) from surgery. The crude cumulative incidence at 5 and 10 years was estimated to be 4.3% and 5.9% respectively. In the T1 group the axillary recurrence rate at 72 months was 4.1% (9/219 patients). Eight patients were managed with radiotherapy, five with axillary dissection and one patient had both treatments. Four patients subsequently had further progression and died from disease and one patient from unrelated conditions. Twenty-three patients developed disseminated disease at a median time of 38 months (range 7–108) from initial surgery. The crude cumulative incidence of distant metastases at 5 and 10 years was estimated to be 6.2% and 13.4% respectively. Fifteen patients were treated with a second-line hormonal treatment, four patients with radiotherapy only and four patients had both treatments. Twenty-one patients died from disease.

Five patients experienced new primary ipsilateral tumour and five contralateral breast carcinoma. The median time from initial surgery was 22 months (8–64 months) for ipsilateral tumour and 25 months (6–39 months) for contralateral tumour. All patients with ipsilateral tumour were treated with total mastectomy whereas wide resection was performed on the five patients with contralateral breast cancer.

Second primary tumours occurred in 13 patients (seven bowel cancers, three gastric cancers, one renal cancer, one vulvar cancer and one endometrial cancer) at a median time from surgery of 37 months (23–127 months).

Total mastectomy was performed only in patients with a large tumour size (>3 cm) and with a concomitant very small breast. The association of these two factors, large tumour size and small breast, enabled us to perform surgery under local anaesthesia without any complication and with patients discharged on the day of surgery.

The distribution of site of recurrence in relationship to tumour size and ER and PgR status is shown in Table II. Figure 1 shows the crude cumulative incidence of local, axillary and distant relapse as a function of time on the total series, and Figure 2 on 219 T1 tumours. Two hundred and forty-four patients did not have any recurrence and 83 (25.8%) died from diseases not related to breast cancer. Cardiovascular diseases were the most common cause of death. The overall 72 month relapse-free survival rate was 76% with a 95% confidence interval (0.75–0.89) (Figure 3).

### Discussion

The data reported here seem to confirm that a strategy consisting in breast conservative surgery and adjuvant tamoxifen may yield results in terms of locoregional relapse rate equivalent to those obtained with more radical surgical approaches (Robertson *et al.*, 1992). The critical issue is whether axillary dissection may be safely omitted in elderly patients with primary breast cancer and clinically negative axillary nodes.

A randomised clinical trial (Fisher *et al.*, 1985) compared, in patients with breast cancer and clinically negative axillary nodes, radical mastectomy with total mastectomy plus delayed axillary dissection only if nodes became clinically involved. The results at 10 years showed that of the 40% of patients in the group without axillary dissection expected to

**Table I** Clinical characteristics of 321 elderly breast cancer patients

| | *No. of cases* | *%* |
|---|---|---|
| Median age 77 years (range 70–92) | | |
| Pathological tumour size | | |
| pT1 | 219 | 68.2 |
| pT2 | 77 | 24.0 |
| pT3 | 1 | 0.3 |
| pT4b | 24 | 7.5 |
| Histological type | | |
| Infiltrating ductal carcinoma | 203 | 63.3 |
| Invasive lobular carcinoma | 60 | 18.7 |
| Intraductal carcinoma | 18 | 5.6 |
| Mucinous carcinoma | 18 | 5.6 |
| Papillary carcinoma | 10 | 3.1 |
| Tubular carcinoma | 6 | 1.9 |
| Medullary carcinoma | 3 | 0.9 |
| *In situ* lobular carcinoma | 3 | 0.9 |
| Receptor status (available in 267 cases) | | |
| $ER^+$ $PgR^+$ | 183 | 68.5 |
| $ER^+$ $PgR^-$ | 56 | 21.0 |
| $ER^-$ $PgR^+$ | 7 | 2.6 |
| $ER^-$ $PgR^-$ | 21 | 7.9 |

**Table II** Patients distribution by pathological tumour size (pT), ER, PgR and site of recurrence

| *Events* | *Pathological stage* *pT1* | | *pT2* | | *pT3* | *pT4b* | | *Total* | | *ER⁺* | *ER⁻* | *Not performed* | *Total* | *PgR⁺* | *PgR⁻* | *Not performed* | *Total* |
|---|---|---|---|---|---|---|---|---|---|---|---|---|---|---|---|---|---|
| Local relapse | 12/219 | 5.4% | 3/77 | 3.9% | 0/1 | 2/24 | 8.3% | 17/321 | 5.3% | 8 | 7 | 2 | 17 | 6 | 9 | 2 | 17 |
| Axillary node involvement | 9/219 | 4.1% | 2/77 | 2.5% | 0/1 | 3/24 | 12.5% | 14/321 | 4.3% | 13 | – | 1 | 14 | 9 | 4 | 1 | 14 |
| Distant metastasis | 8/219 | 3.6% | 13/77 | 16.9% | 0/1 | 2/24 | 8.3% | 23/321 | 7.2% | 18 | 2 | 3 | 23 | 13 | 7 | 3 | 23 |
| Total | 29/219 | 13.2% | 18/77 | 23.3% | 0/1 | 7/24 | 29.1% | 54/321 | 16.8% | 39/239 | 9/28 | 6/54 | 54/321 | 28/190 | 20/77 | 6/54 | 54/321 |

**Figure 1** Crude cumulative incidence for relapses; 321 elderly breast cancer patients without axillary dissection.

**Figure 2** Crude cumulative incidence for relapses; 219 T1 elderly breast cancer patients without axillary dissection.

**Figure 3** Relapse-free survival; 321 elderly breast cancer patients without axillary dissection.

have histologically postive nodes, only 18% required subsequent axillary dissection for the development of clinical involvement. This study also indicated that the overall survival is similar if axillary dissection is performed simultaneously with removal of primary breast cancer or at clinical evidence of axillary relapse. In the present series 14 of the 321 patients (4.3%) experienced an axillary relapse after a median follow-up time of 67 months. The selection of patients without clinical nodal involvement, the high rate of T1 tumours and the possibility that an anti-oestrogenic intervention (i.e. tamoxifen having an inhibitory effect on locoregional tumour growth) may account for this low relapse rate. It is reasonable to think that an older woman with T1N0 breast cancer after a combined therapy of surgery and tamoxifen is more likely to die from intercurrent disease than from progression of breast cancer.

Patients with negative receptors generally considered as being of poor prognosis did not have a higher number of distant relapses compared with $ER^+$ patients. Moreover, no patient with an $ER^-$ tumour had an axillary relapse after a median follow-up time of 67 months, whereas local failure resulted more frequently in $ER^-$ patients (25% *vs* 3.3% in $ER^+$ patients). These data may indicate that $ER^-$ tumours determine a higher rate of local relapse without affecting overall survival and that a subgroup of $ER^-$ and $PgR^-$ or $ER^-$ and $PgR^+$ patients respond to an anti-oestrogenic therapy.

Some authors (Allan *et al*., 1985; Margolese *et al*., 1989) suggested tamoxifen only as an alternative to surgery in elderly patients with breast cancer but controlled clinical trials (Gazet *et al*., 1988; Robertson *et al*., 1988; Bates *et al*., 1991) showed that primary tamoxifen was inadequate to control locoregional disease in more than 40% of the cases. We believe that only patients unfit for surgery or who decline a surgical intervention may be treated by tamoxifen alone as first-line treatment. Clinical evidence from randomised trials suggests that there is an increased risk of women developing endometrial cancer if tamoxifen is taken at a dose of 20 mg daily for long periods (Nayfield *et al*., 1991; Catherino *et al*., 1993). In this study we did not observe an increased risk of endometrial or other types of second cancers related to this age group. Only one woman experienced an endometrial cancer 52 months after breast surgery. We point out that no patient in follow-up had a routine periodical gynaecological assessment such as pelvic ultrasonography or hysteroscopy.

Given the role of surgery as primary therapy another issue has to be addressed. Is wide lumpectomy alone adequate therapy or should radiotherapy be administered after breast-preserving surgery? A recent controlled clinical trial (Veronesi *et al*., 1993) has challenged the usefulness of post-surgical radiotherapy in post-menopausal patients treated with conservative surgery. That study compared quadrantectomy *vs* quadrantectomy plus radiotherapy in patients with breast cancer size < 2.5 cm. and showed, in patients older than 55 years, a local relapse rate of 3.8% in the group without radiotherapy after a median follow-up of 39 months. The results of this study are similar to those of the present paper where, with a median follow-up of 67 months, the local relapse rate after conservative surgery and tamoxifen was 5.3%. The data of these two reports suggests that conservative surgery alone in post-menopausal patients with T1 breast cancer may yield an acceptable local control. Other studies (Gazet *et al*., 1988; Reed *et al*., 1989; Bates *et al*., 1991; Fisher *et al*., 1991) reported a higher rate of local relapse after breast-preserving surgery without adjuvant radiotherapy. The disagreement may be explained by the difference in surgical procedures. Wide lumpectomy or quadrantectomy consists in a more extensive excision, compared with lumpectomy, removing at least 2 cm of normal breast tissue around the tumour with the corresponding portion of overlying skin.

It is very difficult to predict the risk of local recurrence in patients with tumours in macroscopically clear margins. The presence of an extensive intraductal component is generally considered as an important predictor of the risk of local relapse (Harris *et al*., 1985). Another explanation may be the presence in the breast of cancer cells beyond the negative margins of resection and such cases are not identified by the pathologist.

All these data seem to indicate a new operative model for the treatment of T1N0 breast cancer in elderly patients: i.e. conservative surgery without axillary dissection, without post-operative radiotherapy followed by adjuvant tamoxifen.

The advantages of conservative surgery without axillary dissection are numerous. Firstly, the omission of axillary clearance would avoid the morbidity linked to axillary surgery including impairment of arm motion and lymphoedema. Secondly, wide lumpectomy or quadrantectomy without axillary dissection can be performed in a day hospital regimen under local anaesthesia and thirdly, it gives the possibility of reducing the high social costs for age-related cancers.

The best way to resolve the issue of whether elderly patients with T1 breast cancer and without clinical axillary involvement should be recommended surgery with or without axillary dissection would be a randomised prospective trial. Such a trial would have the aim of verifying the necessity of axillary dissection and the impact on disease-free survival.

In conclusion, the treatment of early stage breast cancer in elderly patients has to be re-evaluated in the near future, perhaps restricting axillary node dissection to those patients with clinically involved nodes.

### References

ABBATTISTA A, MARIANI L AND BORACCHI P. (1992). Una procedura SAS per l'analisi descrittiva di dati di sopravvivenza in presenza di rischi competitivi. Proceedings of the Eighth Annual SAS User Group. Parma 21–23. Oct. 1992 pp. 211–226. SAS Institute: Milan.

ALLAN SG, RODGER A, SMYTH JF, LEONARD RCF, CHETTY U AND FORREST AP. (1985). Tamoxifen as primary treatment of breast cancer in elderly or frail patients: a practical management. *Br. Med. J*., **290**, 358.

BATES T, RILEY DL, HOUGHTON J, FALLOWFIELD L AND BAUM M. (1991). Breast cancer in elderly women: A Cancer Research Campaign trial comparing treatment with tamoxifen and optimal surgery with tamoxifen alone. *Br. J. Surg*., **78**, 591–594.

BRADBEER J AND KYNGDON J. (1983). Primary treatment of breast cancer in elderly women with tamoxifen. *Clin. Oncol*., **9**, 31–34.

CATHERINO WH AND JORDAN VC. (1993). A risk-benefit assessment of tamoxifen therapy. *Drug Safety*, **8**, 381–397.

CUMMINGS FJ, GRAY R, DAVIS TE, TORMEY DC, HARRIS JE, FALKSON G AND ARSENAU J. (1985). Adjuvant tamoxifen treatment of elderly women with stage II breast cancer. *Ann. Intern. Med*., **103**, 324–329.

DECARLI A AND LA VECCHIA C. (1989). Cancer mortality in Italy. *Tumori*, **75**, 196–201.

FISHER B AND REDMOND C. (1991). Lumpectomy for breast cancer: an update of the NSABP experience. In: *National Institutes of Health Consensus Development Conference on the Treatment of Early-Stage Breast Cancer*. NCI monographs. No. 11. Government Printing Office: Washington DC.

FISHER B, REDMOND C, FISHER ER, BAUER M, WOLMARK N, WICKERHAM L, DEUTSCH M, MONTAGUE E, MARGOLESE R AND FOSTER R. (1985). Ten year results of a randomised clinical trial comparing radical mastectomy and total mastectomy with or without radiation. *New Engl. J. Med*., **312**, 674–681.

GALANTE E, CERROTTA A, MARTELLI G, DEL PRATO I, MOGLIA D AND PIROMALLI D. (1992). Treatment of breast cancer in elderly women: retrospective analysis of 111 wide lumpectomies performed in a day hospital regimen between 1982 and 1988. *Tumori*, **78**, 111–114.

GAZET JC, MARKOPOULOS CH, FORD HT, COOMBES RC, BLAND JM AND DIXON RC. (1988). Prospective randomised trial of tamoxifen versus surgery in elderly patients with breast cancer. *Lancet*, **1**, 1679–1681.

HARRIS JR, CONNOLLY JL, SCHNITT SJ, CADY B, LOVE S, OSTEEN RT, PATTERSON WB, SHIRLEY R, HELLMAN S, COHEN RB AND SILEN W. (1985). The use of pathologic features in selecting the extent of surgical resection necessary for breast cancer patients treated by primary radiation therapy. *Ann. Surg*., **201**, 164–169.

HELLEBERG A, LUNDGREN B, NORIN T AND SANDER S. (1982). Treatment of early localised breast cancer in elderly patients by tamoxifen. *Br. J. Radiol*., **15**, 511–515.

HERMANEK P AND SABIN LH. (1987). *TNM Classification of Malignant Tumours*. International Union Against Cancer, Springer: Berlin.

HOROBIN JM, PREECE DE, DEWAR JA, WOOD RAB AND CUSCHIERI A. (1991). Long term follow-up of elderly patients with locoregional breast cancer treated with tamoxifen only. *Br. J. Surg.*, **78**, 213–217.

KALBFLEISH JD AND PRENTICE RL. (1980). The statistical analysis of failure time data. John Wiley: New York.

KAPLAN EL AND MEIER P. (1958). Non parametric estimation from incomplete observations. *J. Stat. Ass.*, **53**, 457–481.

MARGOLESE RG AND FOSTER RS. (1989). Tamoxifen as an alternative to surgical resection for selected geriatric patients with primary breast cancer. *Arch. Surg.*, **124**, 548–551.

MARTELLI G, MOGLIA D, BORACCHI P, DEL PRATO I, GALANTE E AND DE PALO G. (1993). Surgical resection plus tamoxifen as treatment of breast cancer in elderly patients: a retrospective study. *Eur. J. Cancer.*, **29**, 2080–2082.

NAYFIELD SG, KARP JE, FORD LG, DORR FA AND KRAMER BS. (1991). Potential role of tamoxifen in the prevention of breast cancer. *J. Natl Cancer Inst.*, **83**, 1450–1459.

PREECE PE, WOOD RAB, MACKIE CR AND CUSCHIERI A. (1982). Tamoxifen as initial sole treatment of localised breast cancer in elderly patients. *Br. Med. J.*, **284**, 869–870.

REED MWR AND MORRISON JM. (1989). Wide local excision as the sole primary treatment in elderly patients with carcinoma of the breast. *Br. J. Surg.*, **76**, 898–900.

ROBERTSON JFR, TODD JH, ELLIS IO, ELSTON CW AND BLAMEY RW. (1988). Comparison of mastectomy with tamoxifen for treating elderly patients with operable breast cancer. *Br. Med. J.*, **297**, 511–514.

ROBERTSON JFR, ELLIS IO, ELSTON CW AND BLAMEY RW. (1992). Mastectomy or tamoxifen as initial therapy for operable breast cancer in elderly patients: 5 years follow-up. *Eur. J. Cancer*, **28**, 908–910.

RONCHI E, GRANATA G, BRIVIO M, CORADINI D, MIODINI P AND DIFRONZO G. (1986). A double-labeling assay for simultaneous estimation and characterization of estrogen and progesterone receptors using radioiodinated estradiol and tritiated org 2058. *Tumori*, **72**, 251–257.

SILVERBERG E AND LUBERA J. (1988). Cancer statistics. *CA (Am. Cancer Soc.)*, **38**, 2–19.

VERONESI U, LUINI A, BERETTA E, BORACCHI P, DEL VECCHIO M, FARANTE G, GALIMBERTI V, MARUBINI E, MEZZANOTTE G, SACCHINI V, SALVADORI B, TANA S AND ZUCALI R. (1990). Conservative treatment of early breast cancer. Long-term results of 1232 cases treated with quadrantectomy, axillary dissection and radiotherapy. *Ann. Surg.*, **211**, 250–259.

VERONESI U, LUINI A, DEL VECCHIO M, GRECO M, GALIMBERTI V, MERSON M, RILKE F, SACCHINI V, SACCOZZI R, SAVIO T, ZUCALI R, ZURRIDA S AND SALVADORI B. (1993). Radiotherapy after breast-preserving surgery in women with localised cancer of the breast. *N. Engl. J. Med.*, **328**, 1587–1591.